# Progress in the study of the correlation between sepsis and intestinal microecology

**DOI:** 10.3389/fcimb.2024.1357178

**Published:** 2024-09-26

**Authors:** Yan-Lin Tao, Jing-Ran Wang, Miao Liu, Ya-Nan Liu, Jin-Qiu Zhang, Yi-Jing Zhou, Shao-wei Li, Shu-Fen Zhu

**Affiliations:** ^1^ Department of Critical Care Medicine, Affiliated Hospital of Inner Mongolia Medical University, Hohhot, Inner Mongolia, China; ^2^ Department of Surgery ICU, Affiliated Hospital of Inner Mongolia Medical University, Hohhot, Inner Mongolia, China; ^3^ Department of Respiratory Medicine, Dingzhou People’s Hospital, Dingzhou, Heibei, China; ^4^ Department of Gastroenterology, Taizhou Hospital of Zhejiang Province affiliated to Wenzhou Medical University, Linhai, Zhejiang, China; ^5^ Physical Examination Center, Affiliated Hospital of Inner Mongolia Medical University, Hohhot, Inner Mongolia, China

**Keywords:** sepsis, gut microbiota, high-throughput sequencing, inflammatory response, immune suppression

## Abstract

Sepsis, a disease with high incidence, mortality, and treatment costs, has a complex interaction with the gut microbiota. With advances in high-throughput sequencing technology, the relationship between sepsis and intestinal dysbiosis has become a new research focus. However, owing to the intricate interplay between critical illness and clinical interventions, it is challenging to establish a causal relationship between sepsis and intestinal microbiota imbalance. In this review, the correlation between intestinal microecology and sepsis was summarized, and new therapies for sepsis intervention based on microecological target therapy were proposed, and the shortcomings of bacterial selection and application timing in clinical practice were addressed. In conclusion, current studies on metabolomics, genomics and other aspects aimed at continuously discovering potential probiotics are all providing theoretical basis for restoring intestinal flora homeostasis for subsequent treatment of sepsis.

## 1 Introduction

Sepsis is a life-threatening syndrome resulting from a dysregulated host response to infection (Singer et al., 2016). This poses significant challenges in critical care medicine worldwide (Salomão et al., 2019; Chiu and Legrand, 2021) and represents a major dilemma in contemporary critical care medicine (Cecconi et al., 2018).

Extensive data indicate that tens of millions of people worldwide are affected by sepsis, with a mortality rate of approximately 22% (Fleischmann-Struzek et al., 2020), and its global incidence continues to increase, posing significant challenges to human health and socioeconomic well-being (Salomão et al., 2019). Sepsis involves multiple systems (Huang et al., 2019), and the gut, as the initiator of multiple organ dysfunction syndrome (MODS), plays a major role in this process (Zhang et al., 2018), with the gut microbiota playing a particularly critical role. Dysbiosis of gut microbiota promotes inflammatory responses through metabolism and immune regulation, leading to distant organ damage (Lelubre and Vincent, 2018; Sun et al., 2020) and participating in the pathogenesis and progression of sepsis (Wang et al., 2019).

Conversely, sepsis disrupts the gut mucosal barrier, causing dysbiosis of the gut microbiota and exacerbating disease. Thus, clarifying the correlation between sepsis and the gut microbiota and improving the host’s microbial structure and function may have practical applications in sepsis prevention and outcomes and could become a potential therapeutic target for sepsis (Keskey et al., 2020) (**Table 1**).

**Table 1:** Details, key insights and shortcomings about sepsis and changes in the gut microbiome.

	key point	signalment	mechanism	Involved factor	challenge	article
**sepsis** ↑↓ **intestinal** **microecology**	Sepsis interacts with the microbiome.	Factors affecting microbiota composition: colonization, reproduction, and death.	Infection leads to multiple organ failure, systemic migration of the flora, and increased virulence of the flora at the primary site.	The diversity of bacterial community decreased, and the genus *Candida*, *Staphylococcus* aureus and *Escherichia coli* became the dominant bacteria.	It is easy to ignore microecological treatment in critically ill patients.	The microbiome and critical illness.
**sepsis** **↓** **intestinal microecology**	Therapeutic interventions that regulate barrier integrity are critical to understanding the role of intestinal barrier integrity in various diseases.	Changes in the microbiome directly or indirectly affect the integrity of the barrier, symbiosis with the intestinal barrier. Impaired barrier integrity can lead to an immune response and lead to a variety of pathological conditions.	The intestinal epithelium is composed of different types of specialized epithelial cells, which secrete antimicrobial peptides through toll-like receptors on the cell surface and nucleotide-bound oligomeric domain-like receptors in the cytoplasm to activate the defense mechanism, and co-regulate the inflammatory response of invaders with immune cells.	Regulation of tight junction integrity: TNF-α stimulates NF-κB, interferon decreases adenosine monophosphate activation protein kinase, thereby inhibiting ZO-1 and occludin expression.	Elucidating the signaling pathways involved in tight junction regulation and identifying new barrier restorers are critical to deciphering novel and effective disease treatments.	Mechanisms regulating intestinal barrier integrity and its pathological implications.
	The intestinal epithelial types with different functions, such as goblet cells, Pan’s cells and M cells, cooperate to maintain intestinal homeostasis and promote immune defense to protect the host.	Intestinal flora has co-evolved with host immunity to tolerate normal luminal flora and recognize potential pathogens to protect intestinal mucosa from harm.	The intestinal epithelia is polarized, expressing innate receptors such as TLR-2 and TIL-3 to detect microorganisms and endogenous danger signals, and secreting chemokines and cytokines to recruit and activate neutrophils and macrophages to guide immune response.	Communication between intestinal microbes and intestinal cells: *Clostridium* stimulates the production of 5-HT by chromaffin cells; The intestinal epithelium secretes chemokines and cytokines to stimulate the production of neutrophils and macrophages.	Little is known about the subsets of various intestinal epithelial cell types.	The Intestinal Epithelium: Central Coordinator of Mucosal Immunity.
	The intestinal barrier plays a central role from SARS-COV-2 infection to the development of viral sepsis.	Impaired intestinal barrier integrity allows luminal bacteria, fungi, and endotoxins to migrate, promoting systemic inflammation and immune activation.	The apoptosis of intestinal epithelial cells increased, the expression of tight junctions, Panes’ cells and submucosal Treg cells decreased, and the intraepithelial CD3 (+) T lymphocytes and pro-inflammatory cytokines increased, promoting bacterial and endotoxin translocation.	Endotoxin increased, fos pathway activated, ischemia and hypoxia, nutrient deficiency, claudins and connexins down-regulated, mitochondrial oxidative stress damage, IL-1β, IL-6, TNF-α gene expression increased.	Further clinical studies are needed to explore the potential positive effects of specific intestinal barrier regulation therapy.	SARS CoV-2-Induced Viral Sepsis: The Role of Gut Barrier Dysfunction.
	Changes in the gut microbiota of critically ill patients indicate that *Enterococcus* can be used as a potential microbial marker for monitoring during ICU stay to predict the development of sepsis.	*Bacteroides*, *Clostridium*, and *Acidobacterium* have increased abundances associated with inflammation. In addition, being female and aging can increase the risk of sepsis.	Microbial diversity is lost and function changes occur in the elderly, and harmful species disrupt metabolic and immune homeostasis in critically ill patients.	There are underlying diseases, there is a long history of taking drugs, there are different lifestyles and diets, and the use of antibiotics to obtain their own different drug-resistant bacterial infections and pathogenic strains.	It is necessary to use molecular biological techniques to clarify the changes of intestinal microbiota and detect the characteristics of drug resistance to evaluate the efficacy.	Gut microbiota profiles in critically ill patients, potential biomarkers and risk variables for sepsis.
	Intestinal leakage and intestinal flora imbalance are inherent features of sepsis. Changing intestinal flora imbalance and reducing intestinal leakage can be the next therapeutic target.	Intestinal fistula syndrome is the root cause of gastrointestinal involvement and the development of sepsis virulence. Blood tests of lipopolysaccharide, diamine oxidase, and 1-beta-D glucan can assess intestinal damage and intestinal microbiota damage.	It is associated with intestinal hypoperfusion, immune cell apoptosis, intestinal neuro-humoral immune response regulation, and systemic inflammatory response leading to intestinal immunodeficiency.	High concentration of PAMPs, emergency junction breakdown of intestinal epithelium, cytokine production in serum, neurohormone disturbance (catecholamines promote the growth of iron-metabolizing bacteria, change the composition of bacteria in the gut) macrophages, mast cell release promote adrenocorticoid-releasing factors, increase in intestinal gram-negative bacteria and individual differences, etc.	We are looking forward to the role of new metagenomics for the identification of virus groups, bacterial groups and microorganisms in future research areas.	The leaky gut and the gut microbiome in sepsis - targets in research and treatment.
	Sepsis leads to multiple organ dysfunction. It is a chronic critical condition characterized by severe immune dysfunction and molecular metabolism. The overall recovery has improved.	Pathogen-associated molecular patterns and injury-associated molecular patterns activate innate immune cells through toll-like receptors and NOD-like receptors on the cell surface to initiate the release of type I interferon and TNF-α, IL-1, IL-6 and other pro-inflammatory cytokines.	Endothelial damage, changes in microcirculation, vasomotor tone, limited movement of cells and nutrients into and out of tissues, and imbalances in the coagulation system and inflammatory and anti-inflammatory signals.	Endothelial cells: increased white blood cell adhesion, pro-coagulant state, vasodilation, loss of barrier function; Microcirculation: local stimulation response is impaired, microthrombus, white blood cells, red blood cell thrombus block the lumen, tissue silver expression, fibrin deposition, anticoagulation mechanism is impaired.	There are uncertainties in the optimal sedation regimen, timing and dose of renal replacement therapy, and hemodynamic management.	Sepsis: pathophysiology and clinical management.
**intestinal microecology** **↓** **Sepsis**	An increase in the proportion of potentially pathogenic bacteria in the gut microbiome or changes in microbiome function can affect the susceptibility of the host to sepsis.	Alterations in the gut microbiome increase susceptibility to sepsis by (a) allowing the expansion of pathogenic gut bacteria, (b) initiating an immunopro-inflammatory response, and (c) reducing the production of beneficial microbial products.	The diversity of intestinal microbes decreased, the abundance of *Enterococcus* increased, the proportion of pathogenic gram-negative bacteria increased, and the decrease of intestinal colonization led to immune dysfunction.	Multidrug-resistant bacteria appeared after the use of broad-spectrum antibiotics. Symbiotic bacteria affect the production of cytokines IL-1β, IL-6 and TNF-α, and the SCFA produced by them up-regulates FOXP3, affects T cell differentiation, inhibits histone deacetylation and reduces pro-inflammatory cytokines regulated by NF-κB, and butyrate reduces tissue oxygen concentration to stabilize HIF-1. Acetate produced by *bifidobacterium* acts on epithelial cells to protect against the effects of intestinal E. coli translocation.	For example, the prognosis of FMT microecological therapy for sepsis is at an early stage, and the donor needs to be carefully screened. Strain selection, timing and optimal route of administration need to be clarified in the hope that new microbiome based therapies can improve sepsis.	The gut microbiome’s role in the development, maintenance, and outcomes of sepsis.
	IL-22 is a pleiotropic cytokine that plays a role in regulating host defense, barrier function, and microbiome metabolism.	The host and intestinal microecology interact and co-evolve over a long period of time, and bacteria adjust their virulence by sensing the surrounding environment and the density signals of the surrounding bacteria, maintaining a balance between the complex immune system and the body.	IL-22 has an antibacterial defense against various infectious agents and can reduce inflammation and susceptibility to secondary bacterial infections.	IL-22 stimulated transport expansion cells and activated Wnt and Notch pathways to inhibit intestinal cell expansion. IL-22 induces the expression of IL-18 in intestinal epithelial cells and promotes the production of chemokines and cytokines to clear pathogens that penetrate the barrier. IL-22 induces the expression of complement C3 gene to kill bacteria and control the spread of bacteria throughout the body.	IL-22 has been clinically shown to promote inflammation and accelerate tumor growth, and is vulnerable to restricted expression of IL-22RA1 epithelial cells in organs, representing only the primary communication channel between the immune system and specific tissue cell types.	9 The role of IL-22 in intestinal health and disease.
	Infection caused by *Pseudomonas aeruginosa* is associated with an immune escape mechanism that increases the survival rate of the bacteria in the host.	The alteration of the symbiotic relationship between opportunistic pathogens and the gut makes them have a stronger pathogenic phenotype, and *Pseudomonas aeruginosa* is a typical example of inducing virulence.	By evading TLR recognition, it interferes with complement system activation, inhibits phagocytosis, interferes with cytokine production, inhibits inflammasome suppression, reduces antigen presentation, interferes with cellular and humoral immunity, and induces apoptotic cell death.	Inhibit the generation of ROS and NET; Escape from antimicrobial peptide LL-37; CFTR inhibitor interferes with antigen delivery to avoid T cell recognition; The loss of mutational function of LasR removes the QS gene from the regulatory system.	*Pseudomonas aeruginosa* still has resistance in the absence of nutritional conditions and the presence of antimicrobials, and the infection control effect is not good.	10Immune escape strategies of Pseudomonas aeruginosa to establish chronic infection.
	*Lactic acid-*producing bacteria can promote the growth and development of intestinal stem cells.	*Bifidobacterium* and *Lactobacillus genera* as lactic acid-producing bacteria significantly increased the expansion of ISC, Panzer’s cells, and goblet cells.	Metabolites that activate mucosal and systemic immunity and resistance to infectious diseases and promote epithelial proliferation in mature intestinal organs.	Lactate and the receptor Gpr81 promote intestinal stem cell-mediated epithelial regeneration by stimulating the Wnt/β-catenin signaling pathway in Paneth and intestinal stromal cells, The expression of Wnt/β-catenin pathway related genes (namely Wnt3, Ctnnb1, Axin2 and GSK3β) was enhanced.	At present, in the stage of prophylactic administration, it is still necessary to screen the dominant strains with high throughput measurement.	Microbiota-Derived Lactate Accelerates Intestinal Stem-Cell-Mediated Epithelial Development.
	Fecal microbiota transplantation is often a method of altering the composition of the gut microbiota during disease and has a restorative effect in the treatment of gastrointestinal diseases caused by pathogenic or conditional pathogenic microbial activity.	Antibiotics can save lives, but they also kill the beneficial microbial communities in organisms and cause harmful bacteria to become resistant to antibiotics.	It acts on the immune response to treat recurrent or refractory *Clostridium* difficile infection when antibiotics are ineffective, and improves the plasma metabolic parameters of metabolic disorders.	Bacterial metabolites such as bile acids and short-chain fatty acids come into direct contact with host metabolite-sensitive receptors expressed by immune cells in intestinal mucosa, leading to mucosal immune response activation and activation of macrophages, dendritic cells and T cells.	The side effects of FMT mainly include abdominal discomfort, abdominal cramps, constipation, etc., which is related to inaccurate analysis of fecal donor materials and chronic disease aggravation of recipients.	Fecal microbiota transplantation in disease therapy.

### 1.1 Sepsis destroys the intestinal mucosal barrier and leads to bacterial disturbance

The intestinal mucosal barrier function (MBF) is an innate defense mechanism that prevents harmful substances and pathogenic bacteria within the intestinal lumen from entering the body. These include mechanical, biological, chemical, and immunological barriers (Subramanian et al., 2020). A well-maintained intestinal mucosal barrier effectively preserves the balance of the intestinal environment (Chelakkot et al., 2018), collaborates with commensal gut microbiota to prevent invasion by pathogenic bacteria, and thus prevents ectopic colonization of extraintestinal organs via the “gut-liver axis.”

In sepsis, the lack of nutrients in the intestine under ischemic and anoxic environments leads to metabolic abnormalities, sulfide and PH increases, intestinal epithelial cell apoptosis, and intestinal villi rupture, affecting the expression of claudins, ligand adhesion molecule A, occlusive protein, and occlusive band-1, as well as the activation of myosin light chain kinase (Zhang et al., 2022). The endotoxin released at the same time binds to toll-like receptor 4 expressed in a variety of cells, such as fos, IRF, mTOR/STAT3, and other pathways; genes that transcript and encode IL1β, IL6, TNF-α, and other cytokines (Assimakopoulos et al., 2022); and mitochondrial DNA released during sepsis, inducing an intestinal inflammatory response through activation of the STING pathway.

Damage to the intestinal mucous layer affects the release of antimicrobial peptides and intestinal choline phosphatase. The integrity of the mucosal barrier is damaged, permeability is increased, and bacterial clearance ability is impaired, eventually leading to an imbalance of tens of thousands of bacteria in the intestinal cavity. Pathogenic strains with high resistance, such as *Proteobacteria* and *Pseudomonas aeruginosa*, are screened, and the ratio of firmicutes/Bacteroidetes decreases, worsening the intestinal damage caused by sepsis (Hu et al., 2019; Zhang et al., 2022; Haussner et al., 2019).

At present, D-lactic acid, diamine oxidase (DAO) (Wang et al., 2023), bacterial endotoxins, and other indicators can be clinically detected to evaluate intestinal mucosal damage and repair damaged intestinal mucosa through epidermal growth factor, and autologous hematopoietic stem cell transplantation has become a new therapeutic target.

#### 1.1.1 The mechanical barrier

As one of the most crucial components of the intestinal mucosal barrier (Allaire et al., 2018), the mechanical barrier is composed of intestinal epithelial cells (IECs) and intercellular tight junctions (Radeva and Waschke, 2018), which regulate the other three barrier types. Thirty-eight-negative kinase 1 can induce the apoptosis of crypt-specific IECs by promoting the phosphorylation of STAT3, the nuclear translocation of p65, and the subsequent release of IL-6 and TNF-α. TNF-α is believed to cause intestinal mucosal injury directly, while IL-6 plays a key role in the persistence of the inflammatory response. Increased production of these pro-inflammatory cytokines leads to the loss of tight junction proteins (Zhou and Verne, 2018; Goossens et al., 2022).

Simultaneously, decreased lactate production by commensal bacteria in the intestinal lumen results in inadequate induction of Wnt3 expression in Paneth and stromal cells via the lactate-specific receptor GPR81, thereby suppressing epithelial stem cell proliferation (Lee et al., 2018). In addition, Ying-Ya et al. suggested that alterations in tight junction protein content in septic mice can disrupt intercellular tight junctions (Cao et al., 2021), leading to increased intestinal permeability.

#### 1.1.2 The biological barrier

The microbial barrier refers to the normal parasitic microbial community within the host intestine that exerts colonization resistance against exogenous bacterial strains (Haak and Wiersinga, 2017). Through long-term coevolution with the host, the gut microbiota and intestinal mucosa form a barrier called the microbial membrane, which is composed of luminal microbiota and mucosal microbiota.

Luminal microbiota mainly consists of aerobic bacteria (*Enterococcus* and *Escherichia coli*), which are easily expelled from the body by peristalsis and are less likely to form colonies. The deeper layers of the luminal microbiota consume free oxygen to maintain an anaerobic environment that is mainly composed of facultative anaerobes (*Bacteroides* and *Streptococcus*).

The mucosal microbiota, represented by *Bifidobacterium* and *Lactobacillus*, are obligate anaerobes that adhere to epithelial cells and inhibit colonization by potential pathogenic microorganisms (PPMOs) (Niu and Chen, 2021). These parasitic bacteria and Pan cells can produce antibacterial bactericins, such as β-defensin, regenerating protein, and S100 protein, which are key components of host defense (Luo and Song, 2021) and mainly exist in the small intestine. They disrupt bacterial cell wall synthesis by perforating the target and inhibiting peptidoglycans. Interaction with ribosomes or tRNA inhibits protein synthesis, disrupts the cell cycle, and directly degrades target cell DNA, thereby achieving an antibacterial effect (Heilbronner et al., 2021). In addition, the surfaces of Gram-positive and Gram-negative bacteria contain teichoic acid and lipopolysaccharide with net-negative charges, while most antimicrobial peptides are cationic peptides, which phagocytose bacteria through electrostatic attraction and osmotic destruction of the membrane structure; they can also enter cells to perform endocytosis and degradation of bacteria through antimicrobial peptide transporters.

Once pathogenic substances in sepsis breach the mechanical barrier of the intestinal mucosa, the internal environment of the gut microbiota is directly affected, which is related to the expression of tight junction proteins during microbial intervention. Conversely, the gut microbiota can release soluble peptides, toxins, and other metabolites to regulate intestinal permeability; maintain mucosal barrier integrity; promote small intestinal villus growth and development; and facilitate neovascularization, providing protection for protective commensal bacteria against PPMOs. In addition, when the microbial barrier is compromised, it can inhibit host intestinal mucosal immune activation (Gasaly et al., 2021), alter cytokine levels (reduced TNF-α and increased IL-10), alter intestinal permeability, decrease the relative abundance of Firmicutes and Bacteroidetes, and lead to the overgrowth of pathogenic bacteria such as *Escherichia coli* and *Enterococcus*.

#### 1.1.3 The mucus barrier

The integrity of the mucus barrier is one of the first protective lines of the gastrointestinal tract and an important part of the intestinal barrier. This multicomponent gel complex can protect intestinal epithelial cells from invasion by intestinal lumen pathogens. It is composed of approximately 30 core proteins, mainly mucin, antimicrobial peptides, and immunoglobulin A. Over the decades of research on intestinal mucus, our understanding of the mucus barrier has transformed from a static lubricant to a dynamic and vital part of the gut ecosystem (Li et al., 2021; Bergstrom and Xia, 2022).

MUC2 synthesized by goblet cells is the most basic skeleton of mucin. The precursor of MUC2 is first folded and glycosylated in the endoplasmic reticulum to form mature MUC2, which is then modified by sialylation in the Golgi apparatus to form large gelatinous mucus and secreted into the intestinal lumen. In sepsis, tissue ischemia, reperfusion, and excessive inflammation produce a large number of reactive oxygen species. This not only directly causes cell damage, but also continuously consumes reduced glutathione, resulting in an increase in oxidized glutathione, causing cellular REDOX imbalance as well as an imbalance of GSH/GSSG ratio, which leads to ER stress, resulting in impairment of mucin synthesis and the intestinal mucus barrier (Wu et al., 2023).

Because antimicrobial peptides have specific and diversified intracellular targets, they can be used to carry drugs to targeted cells for targeted therapy. In recent years, owing to the development of synthetic biology, a large number of modified antimicrobial peptides have the advantage of becoming clinical drugs (Keir et al., 2020; Luo and Song, 2021). Bile has been reported to have a dual regulatory effect on the intestinal mucosal barrier. Bile salt binds to endotoxin, and slgA in bile surrounds bacteria, but bile acid induces IEC apoptosis through its related nuclear receptor expression. MBF is easily destroyed in sepsis accompanied by bile metabolic disorders, and short-chain fatty acids (SCFAs), a metabolite of commensal microbiota, enhance mucin production and release by goblet cells (Kayama et al., 2020). Combined with the abundant secretion of digestive fluids and lysozyme in the intestine, harmful substances are diluted, but at the same time, bacterial structures may also be damaged. Ultimately, the combination of the intestinal mucosal immune barrier and intestinal peristalsis aids in clearing pathogens and substances from the body.

Once intestinal mucosal thickness is reduced, lumen coverage decreased, and the adhesion to intestinal flora rendered insufficient, potential opportunistic pathogen can be displaced through the damaged intestinal lumen barrier (Zhou and Liao, 2021).

#### 1.1.4 The immune barrier

The intestinal mucosal immune barrier consists of gut-associated lymphoid tissue (GALT) and scattered immune cells, which are capable of sensing microorganisms and their metabolites and generating immune responses that collectively influence the host’s ability to respond to infections (Di Tommaso et al., 2021).

During sepsis, GALT activation leads to the production of a specific secretory SIgA, which serves as an intermediate carrier to induce immune maturation, pathogen resistance, and regulation of commensal microbial composition. SIgA enters the intestinal lumen and coats GGram-negative bacteria, forming antigen-antibody complexes. When recognized by intestinal dendritic cells, SIgA induces the release of local IL-10 and TNF-α, further stimulating immune regulatory T-cells and hindering bacterial binding to epithelial cell receptors. It also stimulates intestinal mucus secretion and accelerates the flow of the mucus layer, thereby preventing bacterial adhesion to the intestinal mucosa.

However, under stressful conditions, unlike other antibody isotypes, SIgA remains intact rather than degraded. This allows GALT to exhibit a selective inhibitory state by controlling proinflammatory responses. The local intestinal immune response not only activates humoral and cellular immunity to recognize foreign pathogens and resist invasive microbial pathogens but also prevents disruption of the commensal gut microbiota (Haussner et al., 2019; Jamwal et al., 2020). This illustrates that the cooperative relationship between gut immunity and normal intestinal microecology has evolved to provide mutualistic benefit over time.

### 1.2 Ischemia reperfusion injury

During sepsis, catecholamines are released to redistribute throughout the body to ensure blood supply to the brain, heart, and other important organs. The iron chelation of catecholamines promotes the growth of iron-metabolizing bacteria and changes the composition of bacteria in the intestine (Chancharoenthana et al., 2023). Nutrient metabolism disorders in the body lead to local nutrient deficiency in the intestine and aggravate ischemia and hypoxia after vasoconstriction, causing I/R and accelerating sepsis progression of sepsis (Milano et al., 2020). This process promotes intestinal inflammation and oxidative stress (Wang et al., 2021).

In the serum of animal models of I/R-induced intestinal injury, there is a significant increase in pro-inflammatory cytokines, such as TNF-α and IL-6 (Almoiliqy et al., 2020), as well as abnormal levels of superoxide dismutase and lactate dehydrogenase (Lei et al., 2022). IECs exhibit widespread autophagy and apoptosis, leading to the shedding of intestinal epithelial villi and an increase in intestinal permeability, resulting in the breakdown of the intestinal barrier function (Morelli and Passariello, 2016). This can lead to ectopic colonization of opportunistic pathogenic bacteria in the intestinal lumen and in severe cases may lead to systemic inflammatory response syndrome (SIRS) and MODS (Wang et al., 2021).

In contrast, intestinal epithelial hypoxia promotes the growth of Gram-negative bacteria, such as *P. aeruginosa* and *E. coli*. This may also lead to mitochondrial membrane depolarization, which converts intracellular ATP into hypoxanthine (Delano and Ward, 2016; Yang and Zhang, 2021). During reperfusion, hypoxanthine reacts with O_2_ in the presence of xanthine oxidase, generating cytotoxic superoxide anions that cause damage to intestinal tissues that leads to dysbiosis of the intestinal microbiota (Mahendran et al., 2018).

### 1.3 Sepsis eruption with inflammation and immunosuppression

Sepsis disrupts the mucosal barrier function (MBF), which impairs its ability to suppress pathogenic microorganisms and the pathogen-associated molecular patterns (PAMPs) released by them. PAMPs bind to pattern recognition receptors on the surface of host immune cells, including toll-like receptors (TLRs) and nucleotide-binding oligomerization domain-like receptors (NLRs), leading to the release of pro- and anti-inflammatory cytokines, activation of the coagulation and complement systems, and widespread thrombus formation. This exacerbates microcirculatory disturbances in the intestinal mucosa and activates intestinal epithelial innate lymphocytes (ILCs), weakening innate and acquired immune protection against pathogenic bacterial invasion of the intestines.

**Figure 1:**
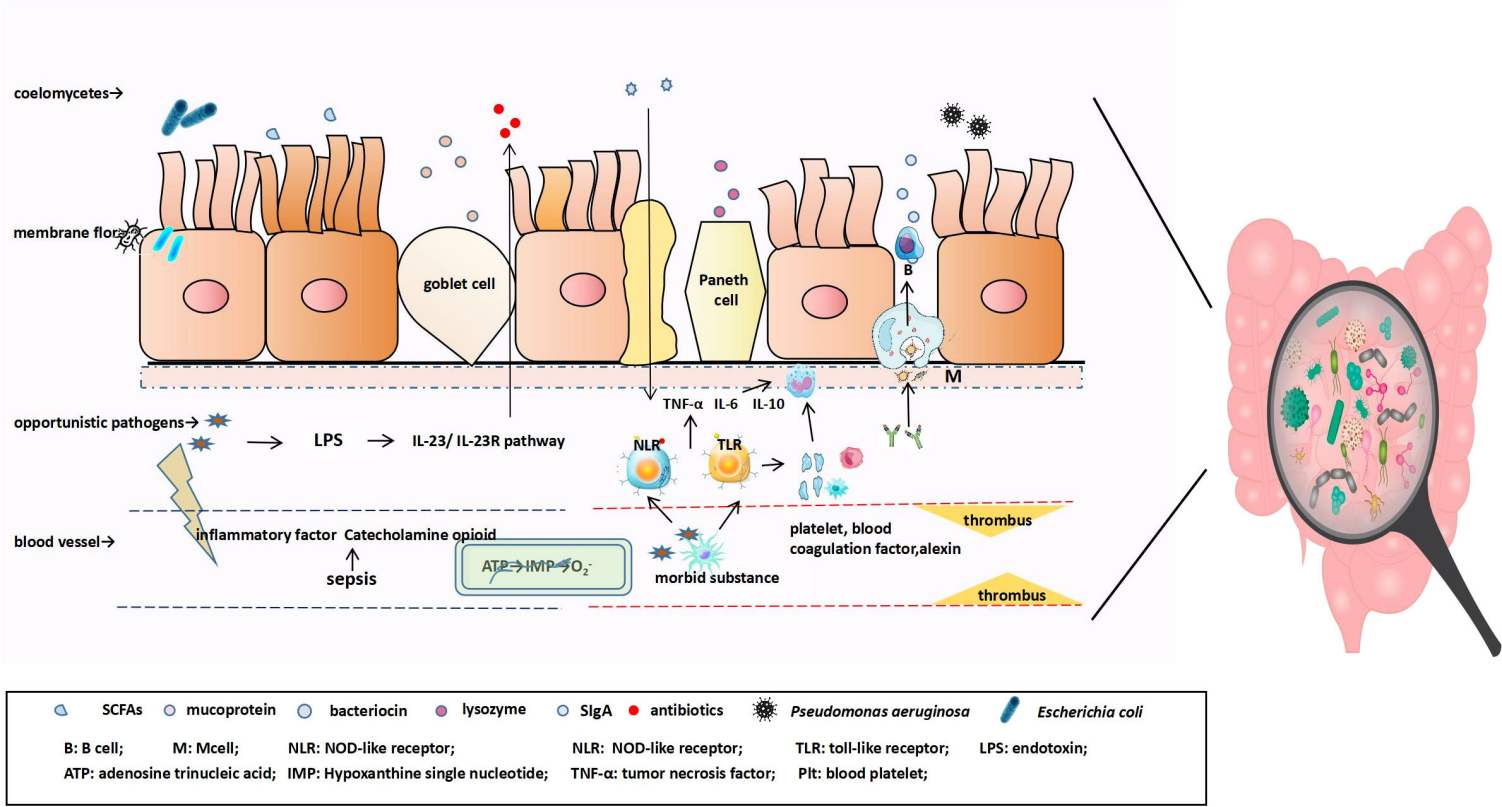
The antigen in the mucosa is transported and presented to antigen-presenting cells (macrophages and dendritic cells), at which point naïve B cells are first activated into IgA+ plasma cells and finally differentiate into IgA-secreting cells that can produce J chain polymers with the help of various factors, followed by further differentiation into IgA-secreting plasma cells. IgA binds to the secretory component SC secreted by intestinal epithelial cells to form SIgA. In the early stage of sepsis, pathogenic substances enter the blood, appropriately activate the inflammatory response, and combine with innate immune cells to produce cytokine IL-22 through the IL-23/IL-23R pathway, promoting the expression of IEC antimicrobial peptides and reducing the number of symbiotic Enterobacteriaceae. As the disease progresses, catecholamines and inflammatory factors are released to directly affect the intestine. Intestinal blood perfusion is reduced, resulting in ischemia and hypoxia, mitochondrial membrane depolarization, and the transformation of intracellular ATP into hypoxanthine. During reperfusion, hypoxanthine reacts with O_2_, under the action of xanthine oxidase, to produce cytotoxic superoxide anions. Pathogen-related molecular patterns bind to TLR and nucleotide-bound oligomeric domain receptors on the surface of host immune cells, inducing the release of pro-inflammatory and anti-inflammatory cellular mediators, triggering the formation of extensive thrombi in the coagulation and complement systems, and aggravating intestinal mucosal microcirculation disorders.

With the progression of sepsis, control is lost over a large number of pro-inflammatory cytokines, inducing SIRS. The latest mouse model of endotoxemia and sepsis induced the molecular cellular process of sepsis, demonstrating that the combination of TNF, IL-18, IFN-γ, and IL-1β can affect gene expression across tissues and reproduce sepsis to a large extent (Takahama et al., 2024). Anti-inflammatory cytokines are then released, and immunosuppression occurs after pro-inflammatory and anti-inflammatory imbalance in the body and excessive release of anti-inflammatory cytokines, which leads to changes in the potential pathogenic bacteria in the intestine and even the diversity of symbiotic probiotics, potentially manifesting as a decrease in the number of obligate anaerobes, consumption of ruminococcus, and an increase in the proportion of proteus and Enterococcus (Delano and Ward, 2016; Liu et al., 2022). Displacement through the lymphatic vessels and blood pathways causes secondary infections (**Figure 1**).

In the early stage of sepsis, when pathogenic substances enter the bloodstream due to infection, a controlled inflammatory response, together with the activation of innate immune cells through the IL-23/IL-23 R pathway, leads to the production of cytokine IL-22. This promotes the expression of antimicrobial peptides by IECs and reduces the population of commensal bacteria from the *Enterobacteriaceae* family, thereby limiting the abnormal proliferation of *Salmonella*. However, if the release of pro-inflammatory cytokines is not controlled, a cascade of inflammatory reactions occurs, activating intestinal epithelial ILCs and increasing IEC permeability. This leads to damage to the intestinal barrier and allows for the colonization of pathogenic bacteria in the intestinal lumen, thereby exacerbating sepsis and potentially leading to MODS. Thus, the outbreak of the inflammatory cascade is considered to be the core of sepsis and involves three pathological (Huang et al., 2019) and physiological changes, such as coagulation abnormalities (Arina and Singer, 2021), complement activation (Yang and Zhang, 2021), and endothelial activation.

After vascular injury, white blood cells and platelets adhere and aggregate to prevent bleeding and maintain vascular integrity. In sepsis, the inflammatory cascade intensifies this local infection response, leading to substance leakage within the blood vessels, tissue edema, and reduced local perfusion, thus further damaging the endothelial barrier. The microbial community within the body establishes connections with platelets and complements to provide protection against the host, thus confirming that a low platelet count is independently related to sepsis-related mortality. In the early stages of sepsis, tissue factor-driven coagulation activation triggers immune defense. During this process, large quantities of coagulation factors and platelets are consumed, leading to disseminated intravascular coagulation (Iba et al., 2019). Experiments have shown that inhibiting tissue factors can suppress the activation of the coagulation system and even prevent MODS and sepsis-related deaths. In addition, inflammation and coagulation are genetically homologous, and coagulation factors (FVIIa and FXa), thrombin, and fibrin activate pro-inflammatory cell signaling through protease-activated receptors (PARs). Notably, complement activation, including C3a and C5a, is part of protective immunity but also exerts potent pro-inflammatory effects (Mollnes and Huber-Lang, 2020). Overactivation can release granulocytic enzymes, increase endothelial permeability, and cause tissue damage and organ failure (Gotts and Matthay, 2016). As microcirculation failure progresses to disseminated intravascular coagulation, platelets, coagulation factors, and other components are further consumed, exacerbating the intestinal injury.

During the progression of sepsis, in addition to pro-inflammatory cytokines, anti-inflammatory factors such as IL-10 and IL-13 are released, which can antagonize the synthesis of TNF-α, IL-1, IL-12, and other pro-inflammatory cytokines (Yang and Zhang, 2021). Excessive release of anti-inflammatory cytokines in the body can lead to pro- and anti-inflammatory imbalance, resulting in an excessive inflammatory response, and the accompanying overwhelming activation of PAMP and damage-associated molecular patterns (DAMPs) from the organism causes the apoptosis of immune cell components such as neutrophils and macrophages, resulting in immunosuppression and progression to the compensatory anti-inflammatory response syndrome stage (van der Poll et al., 2017; Wen et al., 2022).

In sepsis-related immunosuppression, innate and adaptive immune system functions are suppressed, the differentiation ability and number of related immune cells are decreased, and the proportion of inhibitory factors and apoptosis *in vivo* are increased. This reduced immune function and increased susceptibility to infection can lead to systemic infection, resulting in immune imbalance, MODS, or decompensated inflammatory response syndrome. It is an important component of intestinal homeostasis imbalance complicated by secondary infections, leading to death in patients (van Vught et al., 2016).

The innate immune system, also known as innate immunity, has non-specific and rapid response capabilities and participates in adaptive immune responses. Research data indicate that, during immunosuppression, the expression of the peripheral blood monocyte surface antigen (mHLA-DR/CD14+) is downregulated, which inhibits the release of pro-inflammatory cytokines and impairs the ability to recognize and phagocytose endotoxins. Clinical monitoring of changes in mHLA-DR levels in real-time can guide immunomodulatory treatments for patients with sepsis to some extent. Impaired antigen presentation capacity and functional defects suggest a poor prognosis and decreased ability to resist infection (Venet and Monneret, 2018; Huang et al., 2019). Adaptive immune suppression mainly manifests as abnormal differentiation of various subsets of lymphocytes. Rapid screening of lymphocyte counts can help identify an impaired immune function. The main manifestations include a shift in T-cell differentiation toward the TH2 type, an increase in regulatory T cells (Treg cells), and a decrease in γδ T cells, indicating inhibition of the body’s immune function and a negative regulatory effect on sepsis infection (Kayama et al., 2020).

## 2 The disturbance of intestinal flora induces sepsis

**Figure 2:**
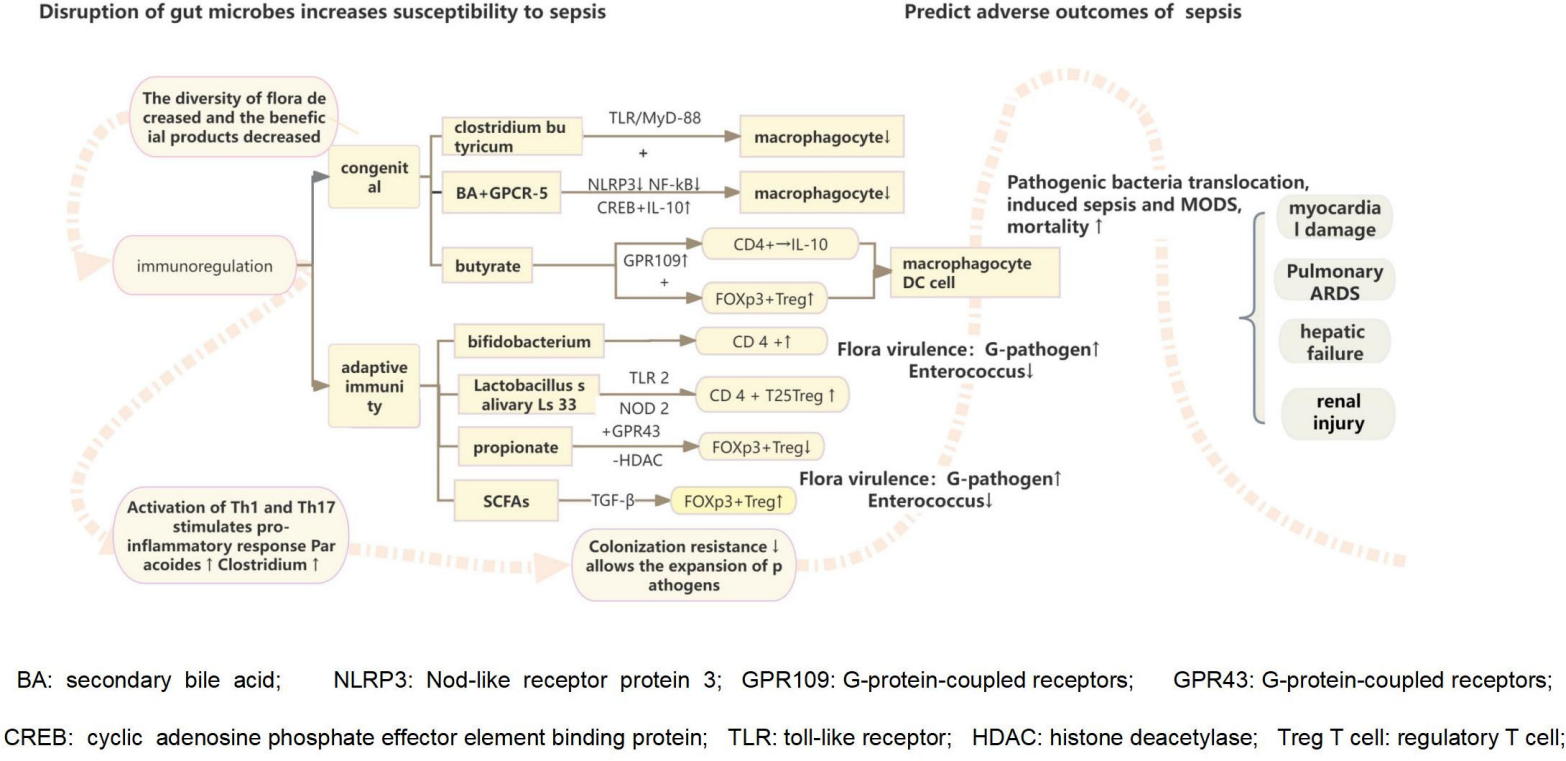
The destruction of intestinal flora reduces the growth and development of intestinal villi, the formation of neovascularization, the ability to shape the intestinal epithelium, and the integrity of the intestinal mucosal barrier function, leading to the colonization and excessive growth of pathogenic microorganisms. Intestinal probiotics also participate in host immune regulation, stimulating related pathways through the flora and metabolites to trigger intestinal innate and adaptive immune cells to respond to intestinal damage. They play a vital role in curbing the colonization and growth of pathogenic microorganisms. The imbalance of intestinal environment combined with the excessive release of inflammatory cell mediators initiates the inflammatory response, which induces an increase of inflammation-related microorganisms in the intestinal cavity, the change of pathogenicity leads to decreased intestinal colonization resistance.

Gut microbiota is a crucial component of the human microbiome (Zheng et al., 2020), and its role in human health and disease has been at the forefront of research. Gut microbiota consists of communities of bacteria, fungi, viruses, and other microorganisms (Kayama et al., 2020). It exists in the environment of the host intestinal tissues, cells, and their metabolites, facilitating independent interactions between substances, energy, and information. The gut microbiota actively participates in regulating the host nutrition (Martin-Gallausiaux et al., 2021), metabolism, inflammation, immune response, and multi-organ function. When the gut microbiota is imbalanced, the associated pathological changes can promote the occurrence and development of sepsis. Disruption of the gut microbiota can lead to the dysregulation of various physiological processes (Adelman et al., 2020), as shown in **Figure 2**.

### 2.1 Gut microbial dysbiosis increases susceptibility to enterogenic sepsis

In the past decade, advances in molecular gene sequencing technology, particularly high-throughput sequencing, have led to significant progress in addressing the limitations of traditional cultivation methods (Wang et al., 2015). These developments have deepened our understanding of gut microbiota interventions in critically ill patients (Shahi et al., 2019), which is crucial for elucidating the pathogenesis of sepsis. Host and intestinal microecology have been interacting for a long time, allowing co-evolution. Bacteria adjust their virulence by sensing the surrounding environment and density signals of the surrounding flora, thereby maintaining a complex immune system balance with the body (Keir et al., 2020).

Many studies have shown that the protection of the enteric environment from the invasion of pathogenic substances mainly involves intestinal symbiotic bacterial homeostasis and interaction with the intestinal mucosal barrier and local intestinal immune system. Finally, inflammatory mediators are released to recognize pathogenic substances, restrict the growth and reproduction of pathogens, and clear them from the body. This indicates that the intestinal flora can predict susceptibility to sepsis (Niu and Chen, 2021). If the symbiotic relationship changes, it results in a more potent pathogenic phenotype. *P. aeruginosa*, an opportunistic pathogen, is a typical virulence inducer (Marzhoseyni et al., 2023) and encodes lipopolysaccharide and type IV virulence factors according to its powerful and complex genes and attaches to target cells to evade TLR recognition. It can also release the CFTR inhibitor cif, block antigen presentation to MHCI, avoid T cell recognition, induce cell apoptosis, and interfere with cytokine production and other functions, leading to fatal infection of the host, causing sepsis and remote organ failure (Adelman et al., 2020). However, in clinical practice, changes in gut microbiota alone cannot fully explain the entire pathological process of sepsis. Furthermore, whether the results of experimental animal studies can be directly applied to humans remains unclear. Therefore, more comprehensive experimental data and a theoretical basis are still needed to support the influence of intestinal bacteria on sepsis, and the microbiome is the core of critical care biology and should be implemented in the precision medicine process of the intensive-care unit (Dickson, 2016).

#### 2.1.1 Reduced diversity of symbiotic flora and beneficial microbial products

The gut is the largest reservoir of bacteria in the human body and comprises five major phyla: Bacteroidetes, Firmicutes (Pushpanathan et al., 2019), Proteobacteria, Actinobacteria, and Verrucomicrobia. These gut bacteria not only produce essential nutrients, such as SCFAs, vitamins, and amino acids, but also secrete chemical compounds, such as sulfides that shape the environment necessary for the growth and proliferation of symbiotic bacteria (Haak and Wiersinga, 2017). Antibiotics are the cornerstone of anti-infection treatment, including the local application of non-absorbable antibiotics such as polymyxin E and amphotericin B and short courses of systemic cephalosporins, but they are also associated with side effects (Luo and Song, 2021), such as: reduced diversity of intestinal flora and affected resistance of intestinal flora to colonization, increasing the susceptibility to opportunistic and nosocomial infections (Lankelma et al., 2017), so that opportunistic pathogens and nosocomial infections expand rapidly, and the resilience of low-diversity communities is limited, allowing pathogens like *Clostridium difficile* and vancomycin-resistant *enterococcus* to grow unrestricted.

Bacteriophages containing antimicrobials are currently considered to have anti-inflammatory and immunomodulatory effects, which are considered to be superior to antibiotics, but the risk of obtaining highly purified bacteriophages and their biological behavior in the course of treatment still needs to be verified for safety (Niu and Chen, 2021). Factors such as a decrease in intestinal microbial diversity and beneficial products of colonized bacteria in the intestinal cavity can lead to a decrease in symbiotic probiotics, like bifidobacteria, and increase the abundance of potential opportunistic bacteria such as *E. coli* to become the dominant bacteria. As a result, intestinal homeostasis is unbalanced, the risk of sepsis is increased, and the mortality of patients with sepsis has increased (Adelman et al., 2020; Niu and Chen, 2021).

Furthermore, this imbalance in the gut microbiota can affect the treatment of sepsis patients during hospitalization and contribute to an increased risk of mortality among sepsis patients.

#### 2.1.2 Initiates a pro-inflammatory response to modulate the immune system

An imbalance in the gut microbiota leads to an increased density of bacteria such as *Proteobacteria*, *Clostridium*, and *Enterococcus* (Agudelo-Ochoa et al., 2020), which are closely associated with inflammation (Skelly et al., 2019). Dysbiosis also induces Th1 and Th17 cell-driven inflammatory responses to protect the host from harmful microbes. In addition, gut microbes adhere to IECs and provide metabolites to maintain the integrity of the gut mucosal barrier. They also release cytokines, which shape the mucosal immune system. This separation from the immune system helps avoid recognition and clearance, leading to immune tolerance. However, it also induces the differentiation of innate and adaptive immune cells, which participate in the clearance of pathogenic substances (Skelly et al., 2019).

Segmented filamentous bacteria are symbiotic bacteria that are found in both mice and rats. They adhere to the IECs of the ileum and induce the production of serum amyloid A. This, in turn, promotes the differentiation of Th17 cells and the production of IL-17 and IL-22, which induce IECs to secrete antimicrobial peptides and Reg3 family proteins, helping to resist bacterial infections in the gut (Kinashi and Hase, 2021).

#### 2.1.3 Loss of ability to contain the expansion of pathogenic enteric bacteria

The intestinal lumen is characterized by a competitive relationship between commensal and pathogenic bacteria (Larabi et al., 2020). When this balance is disrupted, there is a shortage of nutrient supply, competitive colonization sites, and a decrease in SCFAs such as acetate, which allows the growth of potentially pathogenic microorganisms (PPMOs). In addition, this imbalance affects bacteriocin production of bacteriocins (Rodríguez-Sojo et al., 2021), leading to an altered intestinal environment. Furthermore, based on the evaluation of fecal microbiota diversity as a surrogate for colonization resistance using 16S rRNA gene sequencing of rectal swabs from ICU patients, Daniel et al. discovered a strong correlation between decreased colonization resistance, pathogen expansion, and patient mortality rates (Freedberg et al., 2018).

### 2.2 Disturbance of intestinal flora predicts adverse consequences of sepsis

After performing fecal microbiota cultures on sepsis patients, *C. difficile*, *E. coli*, and other corresponding pathogens have been found to be associated with specific nosocomial infections (Freedberg et al., 2018; Adelman et al., 2020). Furthermore, an abnormal proportion of Gram-negative pathogens and *Enterococci* is often an indicator of a higher risk of sepsis in ICU patients, and these potential microbial markers also suggest increased mortality rates (Adelman et al., 2020). Current clinical and experimental evidence indicates that pathogens primarily enter the thoracic duct via the mesenteric lymph nodes and subsequently settle in various organs, including the left subclavian vein (Wang et al., 2019). Colonization can lead to sepsis-induced myocardial damage, acute respiratory distress syndrome, liver failure, and kidney injury via various metabolic and immune pathways. Therefore, changes in specific microbial populations can be assessed to some extent for the clinical management of patients with sepsis.

Existing studies have shown that, by extracting and sequencing the DNA of stool samples from patients with different diseases, a multi-disease diagnosis model based on machine learning can be built for the diagnosis of multiple diseases, which can be used as a noninvasive method for screening various diseases or assessing disease risk in clinical practice (Su et al., 2022). Currently, Random forest machine learning algorithms are used to associate SCFAs, prebiotics, and signature microorganisms that distinguish disease states. Therefore, the target of repairing the intestinal barrier in mice with sepsis has potential clinical applications.

## 3 Microecology-based therapeutic approaches

The migration, growth, colonization, and exit of microbial communities contribute to the balance of microbial ecosystems. Implementing microbial intervention measures in patients with sepsis has great potential for reducing the local bacterial burden and holds promise for both treatment and prevention. The currently proposed approaches include selective digestive decontamination (Myburgh et al., 2022), probiotics (Bauer, 2022), prebiotics, synbiotics, and fecal microbiota transplantation (Antushevich, 2020). These interventions aim to restore or manipulate the microbial composition and function in the gastrointestinal tract, thereby promoting a beneficial microbial balance and potentially improving patient outcomes in the context of sepsis, as shown in **Table 2**.

**Table 2:** Prospects and challenges of microbia-based therapies.

microbiotherapy	prospect	Challenge
SDD	Prevent secondary colonization and overgrowth of potential pathogens.	Selective antibiotic resistance bacteria and persistent antibiotic resistance reservoirs are formed.
Probiotics/prebiotics	Selective probiotic supplementation reduces ventilators associated pneumonia, supports colonization resistance to restore microecological balance, prevents infection by drug-resistant pathogens, and reduces susceptibility to dysecological sepsis.	The effect of selection, mode and time of action of beneficial bacteria on length of hospital stay, mortality and accidental death.
Biostime	Promote the growth of symbiotic bacteria, regulate intestinal microbiota, regulate body immunity, reduce sepsis mortality	The efficacy is greatly affected by individual differences such as environment and dietary habits, and more extensive clinical data are needed to support the efficacy
FMT	It can balance intestinal microecology by deep sequencing, reduce SIRS and immune imbalance symptoms, and induce the restoration of microbial barrier	The conversion of animal experiments to clinical work is scattered, and the detection type, strain gene, pathway of action, therapeutic reproducibility, and safety for patients need to be addressed simultaneously

### 3.1 Mode 1: Selective digestive decontamination

SDD is a strategy that involves the selective eradication of potential pathogens in the gastrointestinal tract using appropriate antibiotics to prevent ventilator-associated pneumonia caused by pathogenic Gram-negative bacteria and overgrowth of upper gastrointestinal yeast. It primarily focuses on reducing the risk of intestinal bacterial translocation by targeting the oropharynx and gastrointestinal tract in critically ill patients (de la Court et al., 2021). However, its use is limited because of the lack of clear evidence regarding its effectiveness. A randomized clinical trial conducted by the Australia and New Zealand Intensive Care Society showed that, in mechanically ventilated critically ill patients, SDD did not significantly reduce in-hospital mortality compared to standard therapy without SDD (Myburgh et al., 2022). Further research is required to determine whether SDD can improve the outcomes of ICU patients with sepsis and whether it contributes to the emergence of antibiotic-resistant bacteria (Cuthbertson, 2018).

### 3.2 Mode 2: Probiotics/prebiotics and synbiotics

The use of beneficial bacteria or their derivatives as alternatives can help alleviate treatment difficulties caused by increased antibiotic resistance and maintain the composition and function of the gut microbiota. The most common approach involves the use of probiotics, prebiotics, or synbiotics. Jumana et al. demonstrated that supplementation of preterm infants with probiotics containing strains of indigenous *Bifidobacterium* and *Lactobacillus* can promote maturation of the gut microbiota and accelerate local intestinal immunity (Samara et al., 2022). In addition, the production of acetate esters by *Bifidobacterium* inhibits toxin production in a mouse model of pathogenic *Escherichia coli* infection, thereby alleviating sepsis-related inflammatory reactions and complications. However, probiotics have not been proven to have long-term beneficial effects and are only effective under certain conditions. This may be because of their potential to induce bacterial infections on their own and the occurrence of side effects when their metabolic products exceed the physiological range of the body. Further research in this area is required.

Prebiotics, including inulin, oligofructose, and oligo-galactose, are dietary fibers that selectively increase the abundance of beneficial probiotic strains, such as *Lactobacilli* and *Bifidobacteria*. Another clinical randomized control trial involving ICU patients with sepsis showed that a synbiotic combination of *Bifidobacterium breve* and *Lactobacillus casei*, along with a prebiotic primarily composed of oligo-galactose (S-HP), can enhance immune responses, increase the abundance of administered strains and other microbial populations, and contribute to maintaining gut microbiota and organic acid levels (Shimizu et al., 2018). However, other studies have indicated that the combination of probiotics and prebiotics does not synergistically improve the intestinal mucosal barrier function (Krumbeck et al., 2018). This discrepancy may be related to inter-individual variation in gut microbiota composition resulting from factors such as race and dietary habits, treatment interventions prior to ICU admission, strain selection, administration methods, and the severity of the individual’s condition affecting the tolerance of synbiotics. Therefore, for a more comprehensive evaluation of the therapeutic effects of synbiotics, clinical research data should be collected to explore their potential mechanisms of action (Brüssow, 2019).

### 3.3 Mode 3: Fecal microbiota transplantation

FMT involves transferring functional microbial communities from the feces of healthy donors into the gastrointestinal tract of a patient to restore a balanced microbiota and provide benefits similar to those of a healthy gut microbiota (Lou et al., 2022). These benefits include enhanced immune responses, an improved gut barrier function, and the prevention of bacterial translocation (Martin-Gallausiaux et al., 2021). This is an emerging targeted therapy for gut microbiota-related conditions (Kim et al., 2020).

The potential advantages of FMT lie in the rapid restoration of a normal gut microbiota through the expression of anti-inflammatory agents, antioxidants, and SCFAs produced by beneficial gut bacteria. Gai et al. (2021) demonstrated that FMT significantly improved inflammatory responses, restored gut microbial diversity, and enhanced the intestinal barrier function in septic mice, suggesting that restoring the benefits associated with the microbial community can reduce sepsis-related mortality to some extent (Gai et al., 2021).

Wurn et al. performed FMT in patients with sepsis associated with steroid and antibiotic use who had persistent diarrhea for 42 days and showed significant relief of SIRS symptoms (Wei et al., 2016). Wei et al. (2016) successfully used donor FMT to treat septic shock, MODS, watery diarrhea, and other symptoms (Li et al., 2015). Thus, FMT seems to have a positive effect on severe and critical illnesses caused by enteroborne infections.

Keskey et al. isolated *Enterococcus faecalis*, *Klebsiella oxytoca*, *Serratia marcescens*, and *Candida albicans* from patients who died of sepsis. FMT with these four pathogens can restore the immune system and alleviate severe infection in animal models of sepsis, which is related to the inhibition of IRF3 expression by the pathogen community and the increase in butyrate-producing bacteria (Keskey et al., 2020).

It is well known that the interaction of chronic pain and anxiety in ICU patients at multiple levels, such as sepsis symptoms, hospital environment factors, and frequent bedside vital signs monitoring and examinations, can lead to sleep disorders, which highlights the importance of sedation and analgesia treatment, which can activate the sympathetic nervous system and hypothalamic-pituitary-adrenal axis. Increased nocturnal cortisol levels inhibit innate and adaptive immune responses (nearly 50% reduction in natural killer cell activity and 50% reduction in lymphokine killer cell activity) (Wang et al., 2020). Dexmedetomidine, an α2-adrenergic receptor agonist, exerts a short-acting calming effect while modulating intestinal microbiota and extrenteral organs via the subphrenovagus nerve. It promoted the enrichment of beneficial intestinal bacteria related to anti-inflammatory effects and found that extraction of dexmedetomidine rat fecal supernatant for FMT was able to replicate the therapeutic effect of dexmedetomidine (Xu et al., 2023), reduce the expression of inflammatory mediators, and increase the phagocytic activity of spleen CD8 T cell macrophages and cells.

The intestinal microbiota has also been shown to play a key role in pain and anxiety (Jiang et al., 2018), which may be related to the fact that intestinal microbiota can regulate pro-inflammatory cytokines (An et al., 2020), chemokines, and hypersensitivity through SCFAs, thereby avoiding the activation of nociceptors to cause pain. Transplantation of fecal microbiota from germ-free mice using diazepam has been shown to significantly improve the comorbidities of pain and anxiety and to reverse the changes in gut microbiota induced by pain stimulation (Wu et al., 2023). However, as Yun Wang discovered, FMT can result in varying degrees of adverse events, including mild reactions, such as abdominal pain and bloating, and even serious adverse events, such as infection and death (Wang et al., 2022). Therefore, in addition to eliminating potential pathogenic bacteria in the donor feces, it is also necessary to ensure that the anaerobic environment, diversity of the bacteria contained in the samples, and post-processing modification of the donor microflora can reduce the risk of FMT treatment to a certain extent, and its biological characteristics must be accurately determined at the right place and time. The completion of colonization and proliferation can maximize the avoidance of elimination and avoid unpredictable side effects. The continuous development of smarter therapies based on nano-coated microbes has become the mainstream, and we expect this precision mediation to become the norm.

## 4 Summary

Sepsis is a complex condition, and clinical interventions, such as stress ulcer prophylaxis with acid-neutralizing agents, sedatives, opioids, and antibiotics, can alter the local colonization of bacteria and their metabolites, which is important for the development and progression of sepsis. However, although restoring microbial balance can provide rapid relief and alleviate disease progression, the safety of microbiota-based therapies has not yet been fully established and carries significant risks. Therefore, clinical efficacy data are required to support these treatments. Currently, apart from the use of antibiotics for infection control and supportive therapy, no effective treatment methods are available. Therefore, we look forward to in-depth research on the interaction between the microbiota and sepsis using advanced sequencing methods. This will help elucidate specific target sites and discover novel treatment approaches with a broad range of effects and targets. Better prevention and targeted treatment of sepsis can be achieved by stabilizing gut microbiota on an anti-inflammatory basis.
